# Expression of Human nPTB Is Limited by Extreme Suboptimal Codon Content

**DOI:** 10.1371/journal.pone.0001801

**Published:** 2008-03-12

**Authors:** Fiona Robinson, Richard J. Jackson, Christopher W. J. Smith

**Affiliations:** Department of Biochemistry, University of Cambridge, Cambridge, United Kingdom; Victor Chang Cardiac Research Institute, Australia

## Abstract

**Background:**

The frequency of synonymous codon usage varies widely between organisms. Suboptimal codon content limits expression of viral, experimental or therapeutic heterologous proteins due to limiting cognate tRNAs. Codon content is therefore often adjusted to match codon bias of the host organism. Codon content also varies between genes within individual mammalian species. However, little attention has been paid to the consequences of codon content upon translation of host proteins.

**Methodology/Principal Findings:**

In comparing the splicing repressor activities of transfected human PTB and its two tissue-restricted paralogs–nPTB and ROD1–we found that the three proteins were expressed at widely varying levels. nPTB was expressed at 1–3% the level of PTB despite similar levels of mRNA expression and 74% amino acid identity. The low nPTB expression was due to the high proportion of codons with A or U at the third codon position, which are suboptimal in human mRNAs. Optimization of the nPTB codon content, akin to the “humanization” of foreign ORFs, allowed efficient translation in vivo and in vitro to levels comparable with PTB. We were then able to demonstrate that all three proteins act as splicing repressors.

**Conclusions/Significance:**

Our results provide a striking illustration of the importance of mRNA codon content in determining levels of protein expression, even within cells of the natural host species.

## Introduction

With 20 amino acids and a termination signal being encoded by 64 different triplet codons, the genetic code contains redundancy. Most amino acids are encoded by sets of two or four codons, which differ in the third, or wobble, position of the codon. Use of synonymous codons is, however, non-random and in most species there are clear preferences for particular synonymous codons, corresponding to the pools of available cognate tRNAs [1]. Consequently heterologous expression of proteins is sometimes prevented because the open reading frame (ORF) has a codon content that diverges too much from the codon preference of the host cells [for example 2], [3]–[9]. The rare AGG and AGA Arg codons in *E. coli* are well known to be problematic for expression of foreign proteins [2]. Another well known example is the Green Fluorescent Protein from the jellyfish, *Aequorea Victoria*, which had to be “humanized” for mammalian expression [10]. Although individual species show clear codon preferences, it has been recognized for more than 20 years that individual mammalian genes can have codon contents that diverge substantially from the average [11]. The principle source of variation is the G+C *vs*. A+T content of codon third positions, which is in turn dictated by large scale genomic organization into G+C rich and light isochores [12], [13]. The average GC content of human third codon positions is 59%, but some human genes are enriched in codons ending in A or T, suggesting that they might face similar barriers to expression as ectopically expressed proteins from an organism with a distinct codon preference. We have encountered this phenomenon while investigating the activities of human Polypyrimidine Tract Binding protein (PTB, also known as PTBp1 and hnRNP-I) and its tissue restricted paralogs, nPTB and ROD1.

PTB is a repressive splicing regulator [reviewed in 14], [15], consisting of four RNA Recognition Motif (RRM) domains, each of which can bind RNA [16]. The optimal RNA binding sites for PTB consist of motifs such as UCUU or CUCUCU in a pyrimidine rich context [17]. Such sequences are often associated with alternatively spliced exons, where they act as splicing silencers (e.g. [17]–[20]). PTB exists as two major isoforms referred to as PTB1 and 4 resulting from alternative splicing of exon 9, which leads to the insertion of an additional 26 amino acids between RRM domains 2 and 3 in PTB4. PTB is expressed in various cell types, but exons that are repressed by PTB need to be spliced under some circumstances. There are various ways in which PTB-mediated repression may be modulated, including the action of antagonistic splicing activators [21]–[23], and substitution of PTB by one of its tissue-restricted paralogs. In addition to the PTB1 and 4 spliced isoforms all mammals possess at least two PTB paralogs that share 70–80% amino acid sequence identify with PTB and a conserved overall domain arrangement, but which are expressed in a restricted number of cell types. Neuronal PTB (nPTB , also known as PTBp2 and brPTB) is expressed at highest levels in neurons, where PTB levels are concomitantly decreased. This switch in PTB paralogs is responsible for switching a subset of neuronal specific alternative splicing events [24], [25]. ROD1 is expressed primarily in hematopoietic cells [26], but as yet there are no reports of its activity as a splicing regulator. A third paralog, smPTB, has splicing repressor activity but is present only in rodents [27]. We have previously found that the PTB1 and PTB4 alternatively spliced isoforms have differential activities upon at least one alternative splicing event [28], [29]. We wished to extend this comparison in experiments involving transient transfection of cultured cell lines with expression constructs for the tissue-restricted human paralogs nPTB and ROD1, as well as the PTB1 and PTB4 spliced isoforms.

To our initial surprise, despite using expression constructs that provided a common promoter, 5′ UTR, N-terminal epitope tag and 3′ UTR, and that differed only by the open reading frame (ORF), the levels of expression of nPTB and ROD1 in a range of cell lines were only 1–3% those of PTB. We found that expression of nPTB and ROD1 was limited by their extremely suboptimal codon content, which was characterized by an enrichment of codons ending in A or U. By effectively “humanizing” the nPTB ORF we were able to obtain expression levels similar to PTB, and were then able to demonstrate that nPTB and ROD1 have splicing repressor activity similar to PTB. Our data highlights the fact that, in addition to its familiar effects upon heterologous expression of foreign proteins, mRNA codon content can have a substantial impact upon protein expression in an intra-species context.

## Materials and Methods

### DNA Constructs

Human PTB1, PTB4, nPTB and ROD1 ORF sequences were used for all constructs. The nPTB* ORF was created synthetically (see below and Supplementary data). The expression vector was created by inserting sequence corresponding to the (His)_6_ and XP tags of the Invitrogen pcDNA3.1/His C vector (Cat. no. V385-20) into the *Kpn* I site of the pCMV-Sport-βgal vector, following removal of the βgal ORF. PTB1, PTB4, nPTB, ROD1 and nPTB* ORFs were then cloned into the *Kpn* I/*Sal* I sites of this expression vector.

The *in vitro* translation vector was created by inserting fourteen nucleotides of the mammalian β-globin 5′ UTR into the *Eco* RI/*Kpn* I sites of pGEM3Z (Promega Cat. no. P2151), providing an adequate distance between the transcription and translation start sites to allow efficient *in vitro* translation. PTB1, PTB4, nPTB, ROD1 and nPTB* ORFs were then cloned into the *Kpn* I/*Sal* I sites of this vector.

The *FAS* splicing reporter construct was kindly provided by J. M. Izquierdo and J. Valcárcel [30], [31]. It contains the human genomic sequence of the Fas receptor from exon 5 to 7, inclusively, under the control of the CMV promoter.

The template for transcription of the nuclease protection probe was created by cloning the sequence common to the expression vectors, from 100 bp upstream of the transcription start site to just 5′ of the PTB ORF, followed by an inverted T7 promoter, into the Eco *RI*/Hinc *II* sites of the pUC19 vector (NEB). The control probe template was similarly constructed from a 300 bp fragment starting 100bp upstream of the transcription start site of the pEGFP-C1 vector.

### Sequence Analysis

Codon frequency tables were generated using the codonfrequency program within the gcg WISCONSIN PACKAGE [32]. Codon usage and GC content comparisons were carried out within Microsoft Excel (version X for Mac) spreadsheets. Codon usage and GC content profiles of an average human ORF were calculated from the frequency of each codon in 10,000 codons, as reported in the draft human genome sequence [33]. Sequence alignments were performed using the ClustalW multisequence alignment program, available on-line from the EMBL-EBI (http://www.ebi.ac.uk/clustalw/) [34].

### Synthesis of the nPTB* ORF

The design of the codon optimised nPTB* was achieved through several rounds of analysis and reassignment of synonymous codons such that the codon usage and GC content were more similar to the average human ORF. G+C content at codon third positions was 64.7% for nPTB*, 86% for PTB, 32% for nPTB and 59% for the “average” human mRNA (59%). A few additional substitutions were made to facilitate cloning, but these did not significantly alter the parameters of interest. An alignment of the nucleotide sequence of the human nPTB ORF and the codon optimised nPTB* is shown in Supplementary data. Oligonucleotides, 70–90 nt in length, overlapping by 20 nt, and corresponding to the entire length of the nPTB* ORF were purchased and used in polymerase chain reactions (PCRs) to generate the full sequence *de novo*.

### Cell Culture Experiments

HEK293, L (mouse fibroblast) and HeLa cells were typically maintained in 10 cm plates in D-MEM (Gibco BRL) supplemented with 10% (v/v) FCS and 5% (v/v) L–glutamine at 37°C, 5% CO_2_. 3 to 5×10^5^ cells were plated in 35 mm diameter wells 24 h prior to transfection in OptiMEMI medium (Invitrogen) with 3 µL LipofectAMINE™reagent (Invitrogen) and 1 µg DNA (plasmids) consisting generally of 200 ng splicing reporter or nuclease protection control plasmid (pEGFP-C1 from Clontech) and 800 ng expression plasmid. For control transfections the latter plasmid was substituted with 800 ng pGEM4Z (Promega). When transfecting less than 800 ng effector plasmid, the remainder of the 800 ng DNA was made up with pGEM4Z. After 6h at 37°C, 5% CO_2_, the transfection medium was replaced with 2 mL usual growth medium. RNA and protein were harvested 48 h following transfection.

### Western Blotting

Cells cultured in 35mm diameter dishes were washed twice with 2 mL PBS and then harvested directly in SDS loading buffer. Equivalent volumes were loaded on 15% SDS-PAGE gels. Standard immunoblotting procedures were followed using the anti-XPress mouse monoclonal antibody (Invitrogen, Cat. no. R910-25) at 1/1000 and the anti-ERK-1 (K2) rabbit polyclonal antibody (Santa Cruz Biotechnology Inc., Cat. no. sc-94) at 1/15,000. Membrane stripping for re-probing was carried out with the Re-Blot Plus Strong Antibody Stripping Solution (Chemicon International Inc., Cat. no. 2504).

### Harvesting RNA from Cultured Cells

For analysis of splicing products, cells cultured in 35 mm diameter dishes were washed twice with 2 mL PBS, then total mRNA was harvested using either TRIReagent (SIGMA), or PURESCRIPT® solutions (Gentra Systems) following manufacturer's instructions. Cytoplasmic RNA was harvested for nuclease protection assays 48 h following transfection of cells. Cells were washed with cold PBS, harvested by scraping, and lysed in 50 mM NaCl, 5 mM MgCl_2_, 2% NP-40, 50 mM Tris, pH 8.3, 10 mM vanadyl ribonucleoside complex for 2 min on ice. Nuclear fractions were pelleted by centrifugation and discarded. The cytoplasmic supernatant was extracted twice with phenol/CHCl_3_ (1∶1) at 65°C, then with CHCl_3_ alone and finally precipitated with NaOAc in ethanol. Any genomic DNA carried over was digested with RQ1 DNase (Promega) for 30 min at 37°C.

### Analysis of FAS Reporter Splicing Products

cDNA was synthesized from 2μg RNA using avian myeloblastosis virus (AMV) reverse transcriptase (RT, Promega) in its commercially supplied buffer with 40 pmol RT primer (5′TTG TGC CCA TTA ACA TCA AGC TTG CAT CGA ATC AGT AG 3′). PCR amplification used 1/20^th^ of the RT reaction as template and 250ng each of the following primers: 5′ GTC GAC GAC ACT TGC TCA AC 3′ and 5′ TTG TGC CCA TTA ACA TC 3′. RT-PCR products were analysed on 2% agarose gels. A digital image of the gel was obtained using a SynGene (www.syngene.com) apparatus and the SynGene GeneSnap software (version 4.00.00). Individual band intensities were quantitated using the SynGene GeneTools software (version 3.00.13). The “peak definition by integration” method was used for quantitation with the “rolling disk” method of background correction. These values were used to calculate the proportion of each product within a single RT-PCR reaction.

### Nuclease Protection Assays

Probes were transcribed with T7 RNA polymerase (prepared in-house) from the *Eco*R I linearised DNA templates described above, incorporating [α^32^P]UTP (GE Biotech) throughout. DNA template was destroyed by RQ1 DNase digestion and the probes purified over a polyacrylamide gel. Nuclease protection assays of cytoplasmic RNA harvested from transfected cultured cells were conducted using the RPAIII kit (Ambion, Cat. no. 1415) as per the manufacturer's instructions, and run on denaturing polyacrylamide gels. Results were quantified with a PhosphorImager and ImageQuaNT software (Molecular Dynamics).

### *In Vitro* Transcription and Translation

Capped mRNAs for *in vitro* translation were transcribed with T7 RNA polymerase from DNA templates linearized with *Hin*d III (PTB1, PTB4, ROD1 and nPTB*) or *Acc* I (nPTB), incorporating trace amounts of [α^32^P]UTP throughout, and the RNAs were isolated and quantitated as described [35]. Translation reactions incorporating [^35^S]methionine were carried out in 50–70% (by vol.) nuclease-treated rabbit reticulocyte lysate (Promega), as previously described [36], [37]. Standard translation conditions included 10 mM creatine phosphate, 50 µg ml^−1^ creatine kinase, 4 mM 2-aminopurine, 100 mM KCl, 0.5 mM MgCl_2_, 0.1 mM of each amino acid except methionine, and 500 µCi ml^−1^ [^35^S]-methionine. Where indicated the lysate was supplemented with calf liver tRNA (Sigma) to a final concentration of 60 µg ml^−1^. In some experiments edeine was added to 1 µM to inhibit further rounds of translation initiation. Translation products were separated on a 15% polyacrlyamide gel and visualised by autoradiography with Hyperfilm βmax (GE) or Biomax film (Kodak). Quantitation was carried out by densitometry of the autoradiograms using Phoretix software.

## Results

### Differential expression of PTB paralogs

**Figure 1:**
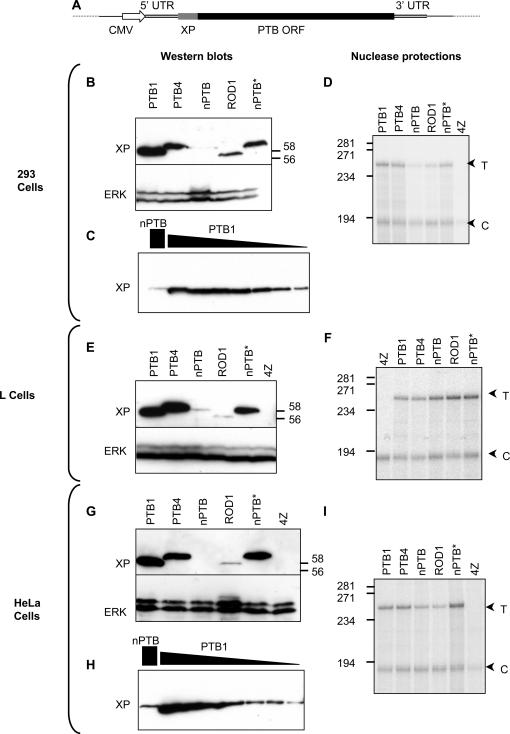
Differential overexpression of PTB, nPTB and ROD1. A) Expression constructs featured a CMV promoter, common 5′ and 3′ UTRs and an N-terminal Xpress epitope tag. Open reading frames (ORF) for PTB and its paralogs were the only variable between constructs. nPTB* is the codon-optimized nPTB expression construct described in the text. B) Western blots of 293 cells transfected with the indicated constructs, probed with anti-Xpress (XP, upper panel) or anti-ERK antibodies. Positions of size markers (kDa) are indicated to the right. C) Western blot comparing the anti-Xpress signal obtained from a dilution series of protein from PTB1 transfected 293 cells (1.0, 0.5, 0.25, 0.062, 0.031 and 0.01 equivalents) with 1.5 equivalents from an nPTB transfection (left hand lane). D) Nuclease protection assay of cytoplasmic RNA harvested from 293 cells transfected with the constructs indicated above. 4Z is a mock transfection (pGEM4Z). “T” is the test probe, corresponding to the Xpress probe region of the expression construct, while “C” is a control probe detecting the cotransfected GFP construct. Positions of size markers (nt) are indicated to the left. E) Western blots of L cells transfected with the indicated constructs, probed with anti-Xpress (XP, upper panel) or anti-ERK antibodies. 4Z is a mock transfection (pGEM4Z). F) Nuclease protection of cytoplasmic RNA from L cells. Details as in “D”. G) Western blots of HeLa cells transfected with the indicated constructs, probed with anti-Xpress (XP, upper panel) or anti-ERK antibodies. H) Western blot comparing the anti-Xpress signal obtained from a dilution series of protein from PTB1 transfected HeLa cells (1.0, 0.5, 0.25, 0.062, 0.031 and 0.01 equivalents) with 1.5 equivalents from an nPTB transfection (left hand lane). I) Nuclease protection of cytoplasmic RNA from HeLa cells. Details as in “D”.

In order to achieve equivalent levels of expression, the open reading frames (ORFs) for human PTB1, PTB4, nPTB and ROD1 were cloned into an expression vector that provided a common CMV promoter, 5′ and 3′ untranslated sequences and an N-terminal Xpress epitope tag (Fig 1A). The constructs were transiently co-transfected into a range of mammalian cell lines along with a GFP expression vector as a transfection control. Unexpectedly, western blotting for the Xpress epitope tag showed much lower expression of nPTB and ROD1 than of PTB1 and PTB4 in all cell lines tested (Fig. 1B,E,G). Reprobing the blots with anti-ERK antibody demonstrated the equivalence of protein loading between samples. Comparison with serial dilutions of total protein lysates from PTB1 transfections indicated that nPTB protein expression levels were 1–3% those of PTB1 in 293 and HeLa cells (Fig 1C,H). The difference in protein expression was evident at various time-points after transfection, arguing against the possibility that the paralogs might be expressed equally but fail to accumulate due to lower stability (data not shown). The possibility that overexpression of nPTB and ROD1 might be toxic to cells was ruled out by cotransfecting PTB, nPTB and ROD1 with a β-galactosidase reporter; no differences in numbers of stained cells were observed (data not shown).

RNA levels were analyzed in parallel by nuclease protection analysis (Fig 1 D,F,I) using a test probe (T) that protects a region corresponding to the Xpress tag present in all transfected constructs, and a control probe (C) that protects a region of the cotransfected GFP, providing an internal control for all experimental samples. Levels of nPTB RNA were comparable to those of PTB1 in L cells and reduced to ∼40% in HeLa cells and to 20% in 293 cells. The relatively modest differences in RNA levels cannot explain the much larger differences in PTB, ROD1 and nPTB protein expression. Indeed, it is likely that the reduced RNA levels may be a downstream consequence of translational stalling (see below). The large variation in protein expression is especially surprising, considering that the amino acid sequences of the four proteins are >74% identical.

### Suboptimal codon contents limit nPTB and ROD1 expression

**Figure 2:**
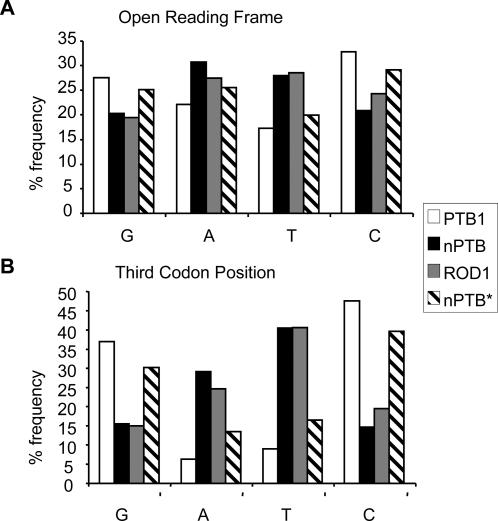
Nucleotide content of human PTB and paralog open reading frames and third codon positions. Histograms show the frequency (%) of the four bases in PTB1, nPTB, ROD1 and the codon-optimized nPTB* for (A) the complete ORFs, and (B) the third codon position.

The expression constructs were identical with the single exception of the ORFs of the proteins being expressed. We analyzed the sequences of the PTB, nPTB and ROD1 ORFs seeking clues to the observed differential expression. PTB expression is negatively autoregulated at the level of splicing [38]. We, therefore, initially searched for sequence motifs corresponding to optimal binding sites for PTB, with the rationale that negative feedback loops operating at the translational level might prevent overexpression of nPTB and ROD1. However, we found no differences in the occurrence of PTB binding sites, or of motifs associated with RNA or protein stability. The one striking difference between nPTB and ROD1 compared to PTB lay in the G+C versus A+T composition of their ORFs (Figure 2). Both nPTB and ROD1 have a lower G+C composition (41.2 and 43.9% respectively) than PTB (60.5%). Moreover, analysis by codon position revealed that this variation in G+C richness was entirely confined to the third codon position (Figure 2B; PTB 86%, ROD1, 35%, nPTB 32%), whereas the first and second codon positions had nearly identical nucleotide composition (not shown). This confinement of the variation to the third codon position is consistent with the high degree of amino acid sequence identity between the three proteins. Consequently, nPTB and ROD1 have a very distinctive codon composition, with a high frequency of codons ending in A or T, while PTB has a much higher proportion of codons ending in G or C. Strikingly, codon bias in the human genome shows an overall preference for G or C in the third codon position (∼59%) [33]. These observations suggested that the differential expression of PTB, ROD1 and nPTB in transfected cells might be due to the enrichment in ROD1 and nPTB of suboptimal codons ending in A and T, which might limit the efficiency with which they are translated.

**Figure 3:**
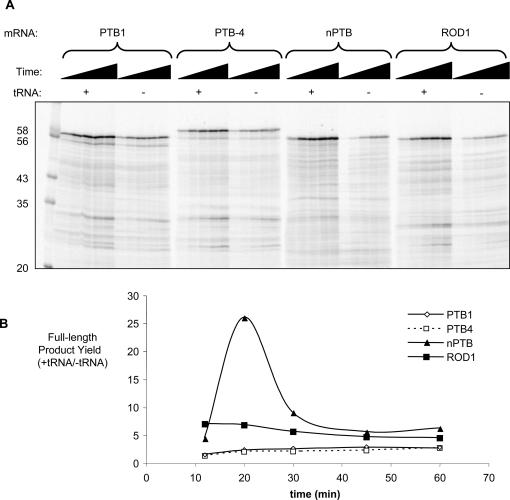
Translation *in vitro* of PTB and paralogs. A) Translation was carried out in rabbit reticulocyte lysate pre-treated with micrococcal nuclease [37] in the presence or absence of additional calf liver tRNA (60 µg ml^−1^) over a time-course of 12, 20, 30, 45 and 60 min, and with 10 µg ml^−1^ of mRNA. Sizes (kDa) of the markers in the left-most lane are indicated to the left of the autoradiogram. B). The effect of tRNA supplementation on relative protein translation in “A” is depicted schematically as the ratio of full-length product produced in the presence and absence of tRNA supplementation.

**Figure 4:**
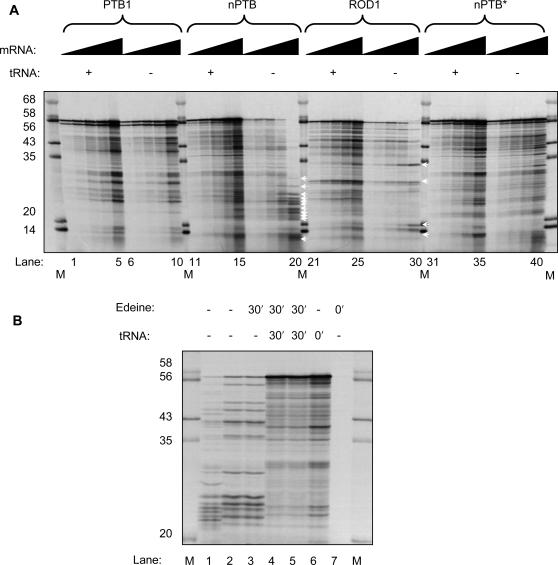
Ribosomal stalling on nPTB mRNA. A) Translation was carried out for 1 hr in the presence and absence of supplementary tRNA, titrating the amount of template mRNA (2.5, 5, 10, 20, 40 µg ml^−1^). Lanes M, protein size markers (sizes in kDa indicated to the left). For clarity only the lane numbers for the lowest and highest concentration of each mRNA are indicated below. Positions of truncated nPTB and ROD1 products are indicated by white arrowheads to the right of lanes 20 and 30. B) Translation of nPTB mRNA (80 µg ml^−1^) was carried out for 60 min except for lane 1, which was stopped at 30 min. Edeine (1 µM) was added at 30 min (lane 3,4,5) or at 0 min (lane 7). Calf liver tRNA (60 µg ml^−1^) was added at 30 min (lane 4 and 5) or at 0 min (lane 6), as indicated. Lanes 4 and 5 are identical replicates. Lanes “M”, molecular size makers. Lanes M, protein size markers (sizes in kDa indicated to the left). Truncated products accumulated in the absence of supplementary tRNA could be chased into full length products by addition of tRNA (compare lane 3 with 4 and 5).

As an initial test of this hypothesis we analysed a 1-hour time-course of *in vitro* translation of PTB1, PTB4, nPTB and ROD1 mRNA in rabbit reticulocyte lysate in the presence or absence of supplementary tRNAs (Fig. 3A,B). Supplementary tRNA is routinely added to reticulocyte lysate with the express purpose of preventing tRNA availability from limiting translation efficiency [37]. ROD1 and nPTB showed a much higher level of induction of translation upon tRNA supplementation than PTB (Fig. 3B) consistent with the hypothesis that their translation is otherwise limited by the availability of cognate tRNAs. Over most of the time-points the levels of induction of nPTB and ROD1 were similar, although at the 20 minute time-point, nPTB translation was activated to a higher degree than ROD1 (Fig 3B). As a further test of this hypothesis, we analysed *in vitro* translation in the presence or absence of supplementary tRNA at a fixed time-point (1 hour) with increasing quantities of mRNAs (Fig 4A). Translational yield was expected initially to increase with mRNA concentration. However, at higher mRNA concentrations some tRNAs might become limiting, leading to a decreased yield of full-length protein in the non-tRNA-supplemented samples. We would also expect to observe the accompanying appearance of truncated protein products associated with ribosomal stalling at codons for which the cognate tRNA is unavailable. Consistent with these predictions, in the absence of tRNA supplementation both nPTB and ROD1 showed a biphasic response to mRNA concentration. An initial increase in yield of full-length product was followed by a decrease, with a concomitant appearance of a number of truncated products (Fig 4A lanes 16–20, 26–30, truncated products indicated by white arrowheads adjacent to lanes 20 and 30). A possible alternative explanation for the appearance of truncated nPTB products is endonuclease cleavage of the nPTB mRNA. However, degradation would be expected to affect a progressively smaller fraction of total mRNA with increasing mRNA concentration. In contrast to nPTB and ROD1, PTB1 translation increased with mRNA concentration and no decrease in yield of full-length product was observed. This is consistent with a higher abundance of the tRNAs required for translation of PTB1. Notably, upon tRNA supplementation, translation of nPTB and ROD1 no longer showed a biphasic response to mRNA titration (lanes 11–15, 21–25).

In order to confirm that the truncated nPTB products observed under conditions of limiting tRNA were due to ribosomal stalling at codons for which the cognate tRNA was limiting, we carried out chase experiments. Truncated products were initially allowed to accumulate, followed by the addition of supplementary tRNA and the translation initiation inhibitor edeine (Fig 4B). Translation for 30 or 60 minutes in the absence of tRNA supplementation showed the expected accumulation of truncated nPTB products (Fig 4B, lanes 1,2). Addition of edeine at 30 min had no effect upon the profile of products at 60 min (lane 3). However, addition of both tRNA and edeine at 30 min led to dramatically increased production of full length nPTB at 60 min (lanes 4 and 5, duplicate samples). Thus, the truncated products formed under conditions of limiting tRNA (lanes 2,3) can be chased into full length products by the addition of tRNA (lanes 4,5). This is consistent with ribosomal stalling at codons for which the cognate tRNA is particularly scarce. This experiment demonstrates that there is no significant “drop-off” of stalled ribosomes. It also rules out the possibility that the truncated proteins are produced from nPTB mRNAs that have been preferentially targeted by endonucleolytic ribonucleases.

### “Humanizing” a human ORF

**Figure 5:**
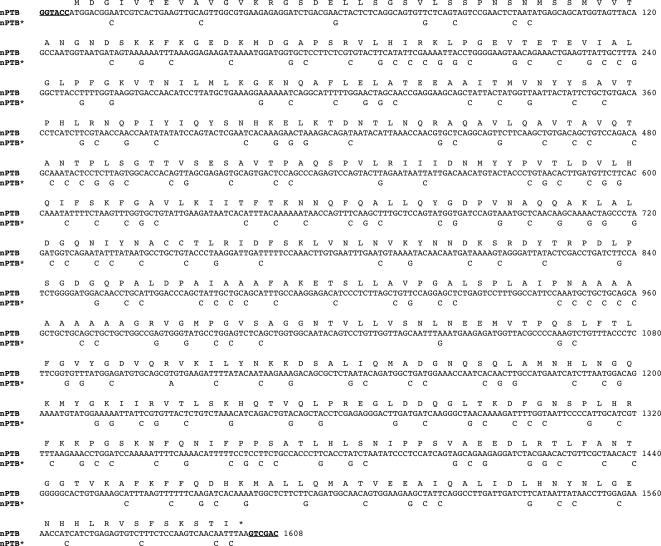
Sequence of nPTB and nPTB* open reading frames. The amino acid sequence is shown on the top line, the human nPTB nucleotide sequence is shown on the second line and the wobble position mutations to create the codon-optimized nPTB* are shown on the third line. The Asp 718 and Sal I sites used for cloning are underlined.

The preceding experiments suggested that lack of expression of human nPTB and ROD1 in transiently transfected human cell lines was related to their suboptimal codon content and the attendant scarcity of required tRNAs. Limitation of expression of foreign proteins in cells of another organism has been observed frequently, and has commonly been circumvented by adapting their codon content to more closely resemble the host organism codon bias [3]–[10]. We, therefore, decided to apply the same principle and constructed a human codon-optimized nPTB ORF (referred to as nPTB*) with 211 silent mutations, encoding an identical nPTB amino acid sequence (Fig 5). The complete nPTB* ORF, and the third codon position in particular, showed a nucleotide composition more similar to that of PTB. The third codon position of nPTB* (64.7% G+C) was closer to the human “average” human mRNA (59%) than either PTB (86%) or nPTB (32%). The codon-optimized nPTB* showed no evidence of tRNA limitation in the *in vitro* mRNA titration (Fig 4A, lanes 31–40). Moreover in all cell types tested, transient transfection of the nPTB* construct resulted in the production of nPTB protein at levels comparable to that of PTB (Fig 1B,E,G). The high level of protein expression from the nPTB* ORF most likely arises from its improved human codon usage profile. An alternative possibility is that the large number of base changes introduced in the construction of nPTB* might have altered secondary structures in the mRNA. However, the vast majority of changes were U to C or A to G, which would be expected to increase the overall stability of secondary structure. Indeed the maximum stabilities of structures predicted by Mfold were −359.7 kcal mol^−1^ for nPTB, but −426.4 kcal mol^−1^ for nPTB*. The only commonly known influence of higher stability secondary structures is decreased translational efficiency when present in the 5′UTR [39], [40], so this explanation for the improved translational yield is unlikely. The simplest explanation consistent with all the data is that the higher expression of nPTB* relative to nPTB is related to its optimized codon content. Taken together, the transfection and *in vitro* translation data (Figs 1,3,4) suggest that poor codon composition restricts nPTB protein expression, even though its mRNA may be present at high levels.

### PTB Paralogs are Splicing Repressors

**Figure 6:**
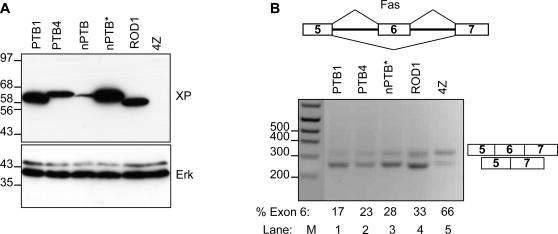
Splicing repressor activity of nPTB and ROD1. A splicing reporter comprising *FAS* exons 5-6-7 [31] was cotransfected with expression constructs for PTB1, PTB4, nPTB* and ROD1 into L cells. PGEM4Z was used in the negative control. *FAS* exon 6 contains a single PTB binding site that mediates exon skipping [31]. A) Western blot probed with anti-Xpress (top) and anti-ERK (lower panel) antibodies. Size markers (kDa) are indicated to the left. Comparable levels of expression were obtained for PTB, ROD1 and nPTB from the codon-optimized nPTB* construct, but not from the original nPTB construct. B) RT-PCR analysis of *FAS* construct splicing. Products were separated by agarose gel electrophoresis. Lane M, size markers (bp), indicated to the left. Lanes 1-5, PTB1, PTB4, nPTB*, ROD1 and pGEM4Z respectively. Numbers immediately below each lane indicate the percentage of spliced product that includes exon 6. Both nPTB* and ROD1 were able to induce similar levels of exon skipping as PTB. Note that nPTB was not included in this panel because the protein is not adequately expressed (panel A).

Having overcome the expression limitations of nPTB we were now able to compare its splicing repressor activity with PTB. *FAS* exon 6 has been shown to respond to overexpressed PTB via a single exon splicing silencer [31]. When transfected into L cells the construct RNA is spliced with predominant inclusion of exon 6 (Fig 6B, lane 5). Overexpression of PTB1 or PTB4 produce a shift towards exon 6 skipping (lanes 1,2). When expressed at levels comparable to PTB1 and PTB4, nPTB, expressed from the nPTB* construct, promoted exon skipping to the same extent as PTB (Fig 6B, lane 3). The original nPTB expression plasmid never had any effects upon cotransfected splicing reporters (data not shown), presumably due to the lack of protein expression (Fig 1, 6A). In this set of experiments ROD1 was also expressed at comparable levels (Fig 6A) and also promoted *FAS* exon 6 skipping to a similar level as PTB and nPTB (Fig 6B, lane 4). These data demonstrate that both human tissue-restricted paralogs of PTB have splicing repressor activity.

## Discussion

It has been known for over 20 years that the codon contents of genes varies widely *within* individual vertebrate species, with the variation being determined by the G+C *vs*. A+T content of codon wobble positions [11]. Nevertheless, it has also been clear that individual species have preferences for particular codons, and that variation in codon preferences *between* species frequently acts as a barrier to experimental over-expression of proteins in host cells of a different species [2]–[9]. As a consequence, many investigators have resorted to “humanizing” the codon content of non-human cDNAs in order to achieve expression in human cells. Despite the recognized variability in codon content of human genes [11], little attention has been focused upon the possibility that expression of human proteins might be limited in human cells by the codon content of their mRNAs. On the contrary, there appears to have been a widespread tacit assumption that even though some human ORFs may have an unusually low G+C content in the wobble positions, the deviation from average is not so extreme as to restrict protein expression due to limiting cognate tRNA availability. Certainly, there seem to have been remarkably few attempts to improve the expression of a mammalian protein in mammalian cells by optimizing wobble positions (reviewed in [41]). In one of the few exceptions, overexpression of human erythropoietin in mammalian cells was shown to be influenced by engineering to contain human (high G+C) or yeast (high A+T) optimized codons, although expression levels differed by only 2.5-fold [42]. Our data demonstrate that inter-gene codon bias can have substantially greater effects upon protein expression within cells of the same organism (Fig 1C,H). Expression levels of the human nPTB protein were 30–100 fold less than those of PTB, or of the codon optimized nPTB* in transfected human cells (Fig 1). RNA levels in the same cells were similar to those of PTB, or only slightly lower, and were insufficient to account for the much larger differences in protein levels. Indeed, it seems plausible that RNA levels may have been slightly depressed as a consequence of translational effects. *In vitro* translation of nPTB showed evidence for translational stalling at discrete positions (Fig 4). Translational stalling has been associated with endonucleolytic mRNA cleavage *in vivo* [43], so the lower amounts of nPTB and ROD1 mRNA compared to PTB in 293 and HeLa cells (Fig 1, C,I) could have resulted from inefficient translation elongation. Our conclusions differ from those of a recent thorough analysis of the effect of ORF G+C composition upon gene expression [41]. In that study, protein and mRNA levels were seen to vary widely with GC content. Translation-level effects were discounted on the basis of comparable *in vitro* translation time-courses of two mRNAs that had 93% and 46% G+C content at third codon positions (compared to 89% and 32% for PTB and nPTB). However, the standard commercially available *in vitro* translation system includes excess tRNAs which can disguise the effects of codon composition upon translation (Fig 3). While many of the cases reported in [41] show large changes in mRNA levels in response to GC content, our observations and others [44], [45] argue that in some cases large translational effects are dominant over relatively minor RNA level effects.

On the basis of established molecular principles our observations are not in themselves surprising, but the magnitude of the effect is remarkable. They suggest that codon content should be an important practical consideration when problems are encountered with protein expression in cell culture, even when the protein and the cells are con-specific. Re-building the ORF, as we have done for nPTB, is a serious undertaking, but it can facilitate lines of investigation that would otherwise have been precluded.

The most plausible explanation for the effect of codon composition upon protein levels is scarcity of the required cognate tRNAs. Various examples of eukaryotic cells with skewed tRNA populations matching the amino acid composition of proteins that are expressed at particularly high levels have been reported [46]–[49]. Therefore, the converse situation is feasible—that cognate tRNA availability might limit expression of an ORF that is enriched in usually rare codons. It is not readily apparent whether there is a specific subset of rare codons that are responsible for the poor translation of nPTB. The *in vitro* translation experiments (Fig 4) showed evidence for translational stalling at up to 14 specific positions. The sizes of these truncated products showed no obvious correlation with clusters of a particular type of rare codon, and it seems likely that codons with U in the wobble positions and those with A were both contributing to the inefficient expression, even if not to the same extent. Systematic identification of problematic codons would probably best be approached by inserting tandem arrays of particular codons into a specific location within an otherwise “normal” human ORF, rather than starting with nPTB in which so many codons appear to be suboptimal.

We overcame the translational impediments of the native human nPTB ORF by *de novo* oligonucleotide based construction of an entire synonymous nPTB* ORF. In order to maximize the chances of success, we did not try to identify particular problem codons, but adjusted the entire ORF so that it was closer in composition to the human average. Of the 211 silent mutations 47% were U to C, 22% A to G, 17% A to C and 13% U to G. Thus, 60% of the mutations removed U from the wobble position and the remainder removed an A. For every synonymous NNA/G codon pair, nPTB shows a higher proportion of codons ending in A compared to both PTB and the human average. The highest enrichment of NNA over NNG codons in nPTB compared with the human average, is in the codons for Leu CTA/G (4.1 fold), Arg CGA/G (2.8 fold), Val GTA/G (2.3 fold) and Gln CAA/G (1.9 fold). For the decoding of NNG/A codons, a tRNA with C in the wobble positions reads only NNG, whereas a tRNA with a modified U in this position decodes NNA and may also be capable of reading NNG, depending upon the exact modification. Thus, there could well be a higher concentration of tRNAs cognate for NNG than for NNA, and this concentration difference could be the complete explanation of the beneficial effect of the A to G mutations. By contrast, all tRNAs cognate for NNC can also read NNU [33], [50], irrespective of whether the tRNA wobble base is G or I. Therefore, any improvement in translation resulting from the 47% U to C mutations cannot be due to differential tRNA concentrations, but must be due to a greater efficacy of the tRNA in reading NNC as opposed to NNU. In fact, it as been shown that bacterial aminoacyl-tRNAs with G at the first antcodon position bind more rapidly to ribosomes programmed with codons ending in C than in U [51], [52].

Our experiments were based upon overexpression by transient transfection, and *in vitro* translation. The extent to which codon bias influences gene expression *in vivo* remains an open question. One possibility is that codon content presents a uniform restriction to nPTB expression across various cell types. Alternatively, cell-type specific variations in tRNA availability might lead to differential translatability, possibly in neurons. The possibility that variations in codon usage might be associated with expression in different human tissues has been suggested [53]. Indeed, papillomavirus L1 protein is translated in differentiated but not undifferentiated keratinocytes despite similar levels of mRNA, and this has been suggested to be explained by differential tRNA populations [45]. However, only 2.3% of synonymous codon variability can be accounted for by tissue-specific expression; the major influence upon codon variability is genome isochore structure [1], [54]. Introns within the nPTB and ROD1 genes have a much higher A+T content than PTB introns, consistent with the location of these genes in G+C light isochores. Indeed, this genomic arrangement is conserved in the murine nPTB and ROD1 orthologs, which show very similar suboptimal codon compositions. The unusual codon content that is imposed upon nPTB by its location in a low G+C isochore could act as a uniform constraint upon translation in various cell types. An alternative, tantalizing, possibility is that cell-specific tRNA populations may modulate the translatability of nPTB. Investigating whether cell-type specific pools of tRNA modulate nPTB translation would first require the identification of cell line(s) that show an altered hierarchy expression from transfected PTB, nPTB and ROD1 constructs. However, all cell lines that we tested showed the same hierarchy of expression (Fig 1). It is possible that this line of investigation could be pursued in a suitable transfectable neuronal cell line, which might show enhanced nPTB translation. In the absence of transfection of constructs that differ only in the PTB/nPTB ORFs it would be difficult to disentangle nPTB regulation by tRNA availability from the numerous other levels at which its expression is regulated (see below).

Having engineered the nPTB* ORF, we were able to demonstrate by cotransfection that nPTB has similar repressor activity as PTB upon *FAS* exon 6. *In vitro* assays with an α-tropomyosin pre-mRNA substrate have also indicated that nPTB has repressor activity similar to PTB [29]. In addition, the nPTB* construct has subsequently been used to complement the effects of PTB and nPTB knockdown upon model actinin and PTB exon 11 reporter constructs [55]. Therefore despite the fact that nPTB is less repressive than PTB upon the *SRC* N1 exon [56], for other splicing events it has repressor activity similar to PTB. This is consistent with recent findings that replacement of PTB by nPTB in neurons has differential effects on large numbers of neuronally regulated exons [24], [25]. While the role of nPTB in the regulation of neuronal alternative splicing has been investigated, to our knowledge this is the first demonstration that ROD1 also acts as a splicing repressor. The codon composition of ROD1 mRNA is slightly less extreme than that of nPTB, and ROD1 expression was more variable between experiments (Figs 1,6). While nPTB expression was uniformly low in all experiments, in some cases we obtained high expression levels of ROD1 and we were able to test its splicing regulatory activity (Fig 6). We therefore did not construct a codon-optimized ROD1 vector. However, alternative splicing is a major contributor to generating protein isoform diversity in the immune system [57], and ROD1 may contribute to this process, perhaps having variable activity upon a subset of hematopoietic alternative splicing events. A reliable codon-optimized ROD1 construct might prove useful in the future investigation of its functions.

Expression of nPTB protein is controlled at a number of post-transcriptional levels. Even though it is transcribed in cells other than neurons, a PTB-induced exon skipping event leads to Nonsense Mediated Decay of the bulk of nPTB mRNA in non-neuronal cells [24], [25]. In addition, it is subject to miRNA mediated translational repression [58]. Both these mechanisms restrict nPTB protein to a narrower range of cell types than nPTB mRNA. Translational control by codon bias, as described here, adds a further layer to this complex arrangement of post-transcriptional control.

As a final comment, it is perhaps worth noting that our references to the “suboptimal” codon content of nPTB mRNA are very much from the perspective of the experimental investigator trying to overexpress a protein. While unusual, the codon composition of both nPTB and ROD1 may, in fact, be optimized for appropriate regulated protein expression.
